# The Discovery of Porcelain

**Published:** 1882-07

**Authors:** 


### THE DISCOVERY OF PORCELAIN.

Kaolin, a natural hydrated silicate of alumina, is absolutely refractory and opaque ; it constitutes the resistant part of porcelain. Feldspars are silicates of alumina and potassa, fusible at a very high temperature into a beautiful transparent glass. If, now, we mix a quantity of feldspar and kaolin, cover the mixture with a layer of feldspar, and heat the whole at a very high temperature, the feldspar will melt and communicate to the opaque clay a clearness greater or less according to the quantity of it present, and to the superficial part of it that beautiful glaze with which all are familiar. A part of the action in this process is chemical, and consists in the production of a new crystalline silicate formed by a combination of all the substances present. The discovery of porcelain in China is traced back to a high antiquity. The Chinese have certainly made it regularly for at least a thousand years ; many authors fix the discovery at fifteen hundred or eighteen hundred years ago, but no evidence exists to justify our going further back than a thousand years. The first pieces that came to Europe were probably brought by the Venetians at the end of the thirteenth century. Charles VII., King of France, received a present of Chinese porcelains, about the middle of the fifteenth century, from the Sultan of Babylon ; but it was not till the sixteenth century that the importation of these Oriental products by Portuguese and Dutch merchants assumed a real importance. The discovery of tender porcelain was made in France toward the end of the seventeenth century, but whether by Louis Poterat or by Reverend, at Paris or Rouen, is disputed. This ware has no relation with real porcelain ; it contains neither kaolin nor feldspar, but is an artificial product, a kind of glass made from a mixture composed essentially of sand, lime, potash, soda, and a small quantity of marine marl. This mixture, made plastic by the addition of manganese or other fluxes, is baked without glazing, and is covered after baking with a glazing composed of silica, lead, potash, and soda. The beauty of the material, its perfect glaze, and the facility with which vitrifiable colors are fixed in it, make of tender porcelain a ware exceptionally adapted to decoration.—*From "Porcelain and the Art of its Production," by* CHARLES LAUTH, *in Popular Science Monthly for July.*

